# Supplementary material for: Inferring transportation mode from smartphone sensors: Evaluating the potential of Wi-Fi and Bluetooth
Source: PLoS One. 2020 Jul 2;15(7):e0234003. doi: 10.1371/journal.pone.0234003 (PMC7332005; doi:10.1371/journal.pone.0234003)
Supplement: S1 Appendix — This appendix contains information about our machine learning approach. (PDF) [file pone.0234003.s001.pdf]

Classification models

Random forest (RF) We fixed the number of trees to 100.[1] For computing the feature importance we used Gini Impurity [1]. When searching for hyperparameters we used from the default settings [2] by computing: maximal depth of the trees as square root of the feature count; the model performance measured using from Gini impurity. The model hyperparameters are:

- maximal tree depth chosen from $\{1, 2, 4, \infty\}$;
- minimum number of sample splits from $\{2, 5, 15\}$.

Logistic regression (RF) We use a multinomial logistic regression for classification. As we rely on RFE we use L2 regularization to reduce size of parameters. For computing the feature contribution the size of the parameters was used as they have been standardized. The model hyperparameters are:

- L2 penalty from $\{10^{-4}, 10^{-3}, ..., 10^{4}\}$.

Support vector machine (SVM) We use an SVM classifier with a linear kernel. We only use hyperparameters for the linear kernel which are limited to L2 regularization:

- L2 penalty from $\{10^{-4}, 10^{-2}, 10, 10^{2}, 10^{4}\}$.

References

1. Breiman L. Classification and regression trees. Routledge; 2017.
2. *scikit-learn* developers. Scikit-learn v 0.20 documentation;.

[1] We also tried to increase the number of trees but it had no discernible effect.
