# Supplementary material for: Inferring transportation mode from smartphone sensors: Evaluating the potential of Wi-Fi and Bluetooth
Source: PLoS One. 2020 Jul 2;15(7):e0234003. doi: 10.1371/journal.pone.0234003 (PMC7332005; doi:10.1371/journal.pone.0234003)
Supplement: S2 Appendix — This appendix contains details of the analysis using other classification models and other transportation modes. (PDF) [file pone.0234003.s002.pdf]

Auxiliary results

Other classification models

We begin with comparing the three different classification approaches: Random Forest (RF); Logistic Regression (LR), and; Support Vector Machine (SVM). The comparison is carried out using the full set of features. The performance in terms of F_1 accuracy and overall accuracy is shown in Figure 1. We find that the random forest (RF) classification model outperforms the two other machine learning approaches, both in terms of accuracy and F1 score. Upon comparing the model output for each of the 1 000 resample iterations we reject the null hypothesis that RF and each of the other models have equal performance (respectively $p = 0.000$ and $p = 0.000$ for LR and SVM). A breakdown of the F1 score into precision and recall can be found in S2 Table.

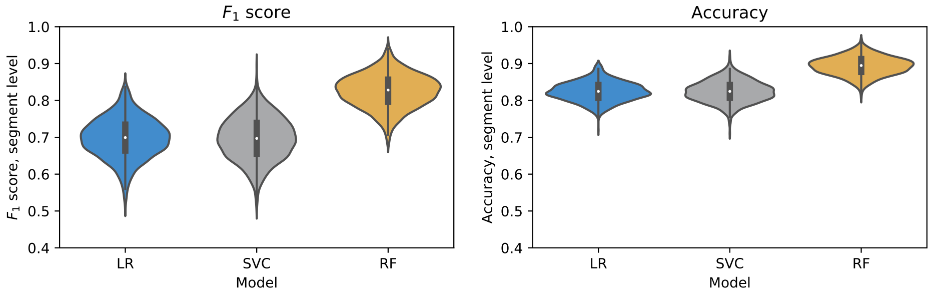

Fig 1. Overall performance of models.

This figure shows violin plots of the test set performance associated with each of the three classification models {LR,RFC,SVM}. The F_1 score is computed as unweighted average across modes. The performance is measured using segment level predictions. The target is "Car vs. Public vs Self-powered" and all features are used. The plots are based on resampling the data 1,000 times into training data for model building and test data for evaluating the model.

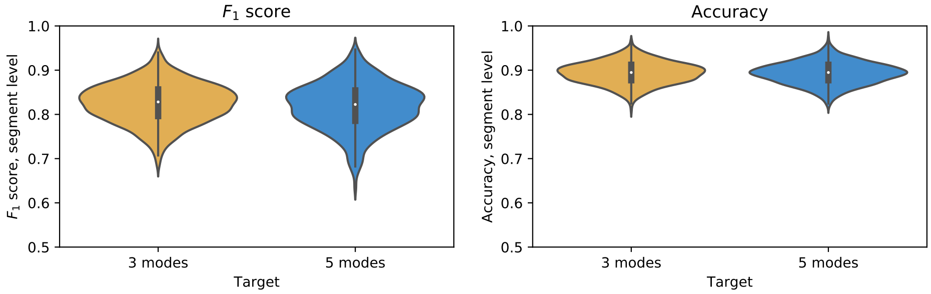

Fig 2. Performance for varying set of transportation modes.

This figure presents violin plots of the test set accuracy associated using the three sets of transportation modes. In each model all the features are used. The performance is measured using segment level predictions. The plots are made from resampling the data 1,000 times into training data for model building and test data for evaluating the model.

Other transportation mode categories

In this appendix we focus extend the analysis to one alternative sets of transportation modes. The first is less detailed and contains only {Motorized, Self-powered}. The

second is more detailed and has five modes in total. The difference from relative to the main set of transportation modes is that public transportation is further broken down into {Bus, Metro, Train}. We call these two models respectively the 2-class target and 5-class target; we call the main set of transportation modes the 3-class target. The F_1 score and overall accuracy of models using the different targets is found in Figure 2.

In Figure 3 we compare the model with and without Wi-Fi and Bluetooth as well features to gauge how these features affect the model performance. The associated mean differences and their tests are found in the text

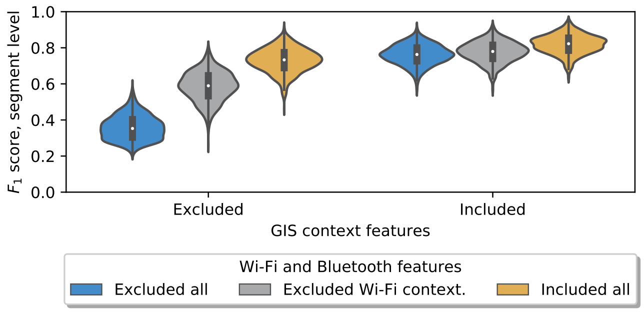

Fig 3. Contribution of Wi-Fi context information to model performance using 5 modes.

This figure presents the model performance for the 5-class transportation mode under various feature sets. The performance is measured using segment level predictions. The plots are made from resampling the data 1,000 times into training data for model building and test data for evaluating the model.

Alternative sampling rate

We conducted an experiment where we changed the temporal resolution from 1 to 5 minutes. We aggregated the temporal resolution by taking the median of numeric features and the mode of categorical features. Below we show the mean segment level statistics under the various features sets estimated using RFC.

GIS context	Wi-Fi and Bluetooth	F_1 score	Accuracy	Precision	Recall
Excluded	Excluded Wi-Fi context.	0.756	0.819	0.795	0.734
	Excluded all	0.617	0.730	0.649	0.604
	Included all	0.822	0.863	0.847	0.809
Included	Excluded Wi-Fi context.	0.804	0.851	0.827	0.789
	Excluded all	0.776	0.830	0.807	0.757
	Included all	0.851	0.883	0.870	0.840

Table 1. Segment level model performance

This table contains all the mean performance measures under various feature sets of including/excluding GIS features as well as including/excluding Wi-Fi and Bluetooth based measures. The performance is measured using segment level predictions. The mean is computed using the performance measures associated with each of the 50 resamples of the data. This table corresponds to Table 4 in the main text.

We show the distribution F_1 scores in Figure 4. When comparing the model with all features against the models where respectively the 'Wi-Fi context' as well as 'Wi-Fi and Bluetooth' features are removed results in a drop in performance of 0.029 ($p = 0.076$) and 0.042 ($p = 0.065$) when GIS features are included. When the GIS features are excluded the drops are respectively 0.080 ($p = 0.000$) and 0.197 ($p = 0.000$) for 'Wi-Fi

context' and 'Wi-Fi and Bluetooth'. This shows that our results gives the same conclusions, but the conclusions are only borderline statistically significant.

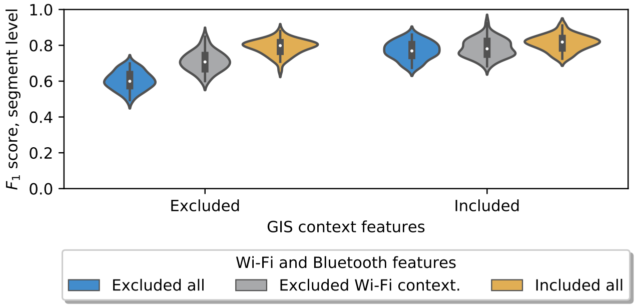

Fig 4. Contribution from Wi-Fi related features on model performance, using 5 minute sampling rate.

This figure presents the model performance for the 3-class transportation mode under various feature sets. The performance is measured using segment level predictions. The plots are made from resampling the data 50 times into training data for model building and test data for evaluating the model.
