# Supplementary figures and images for: Inferring transportation mode from smartphone sensors: Evaluating the potential of Wi-Fi and Bluetooth
Source: PLoS One. 2020 Jul 2;15(7):e0234003. doi: 10.1371/journal.pone.0234003 (PMC7332005; doi:10.1371/journal.pone.0234003)

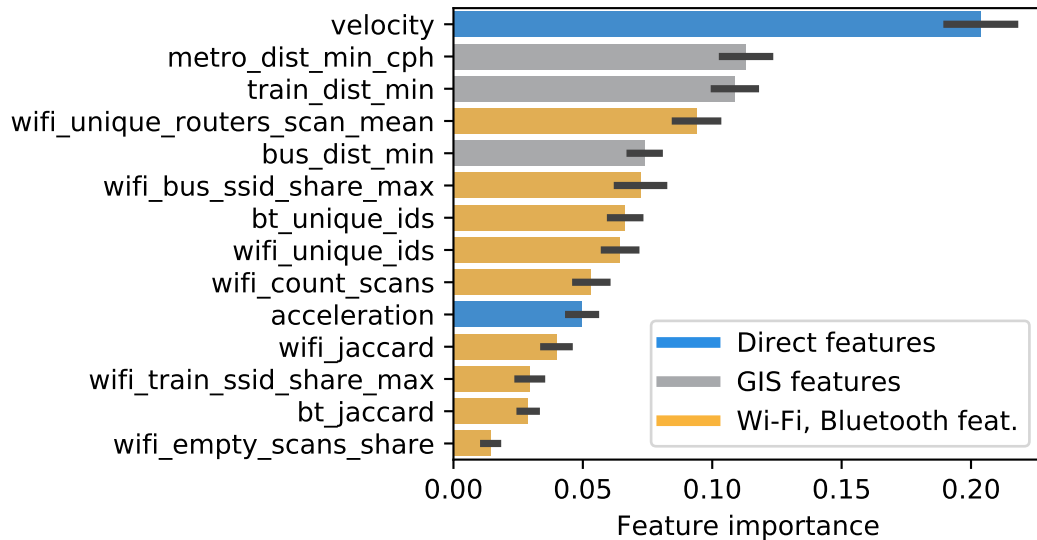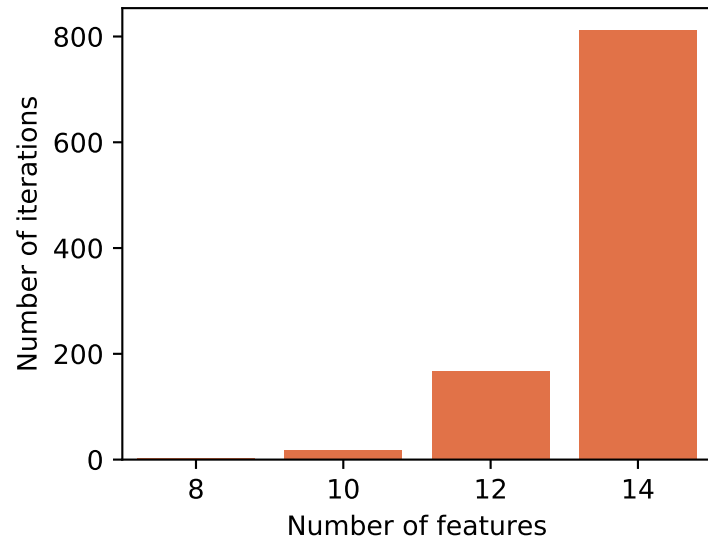

Supplement: S1 Fig — These plots show the feature importance and number of features chosen for the optimal selected models for the 1,000 resampled models. We consider the random forest model, with all features; the target is “Car vs. Public vs. Self-powered”. The black lines in the feature importance plots denote 95% confidence intervals based on resampled data. Feature importance values are computed for each random forest model at the temporal unit of observation (i.e. minute level) using Gini Impurity [79]. Note that feature importance is only for the random forest model with all features. The plots are created by resampling the data 1,000 times into training data for model building and test data for evaluating the model. Direct features are shown in blue, GIS features are shown in gray, and features based on Wi-Fi are displayed in yellow. (PDF) [file pone.0234003.s003.pdf]

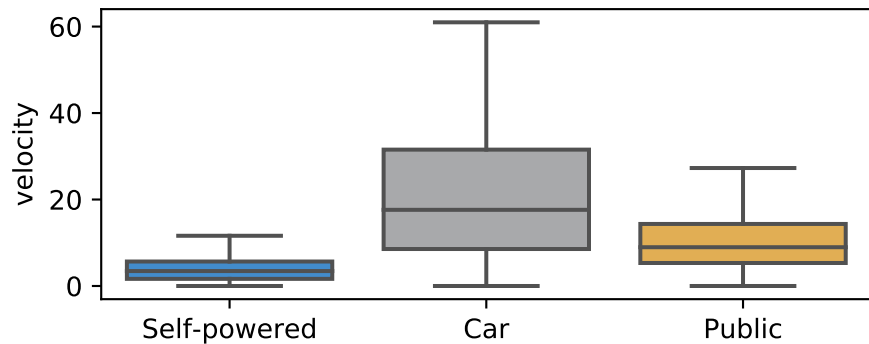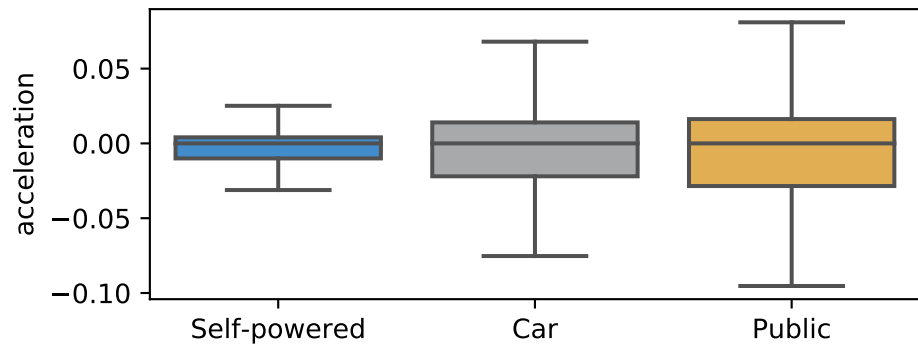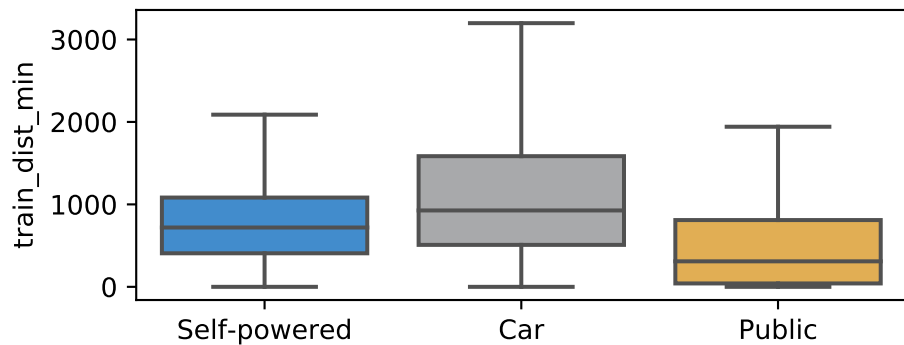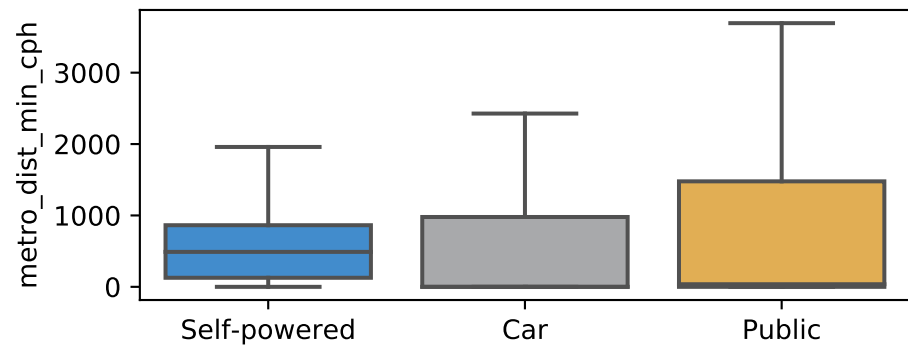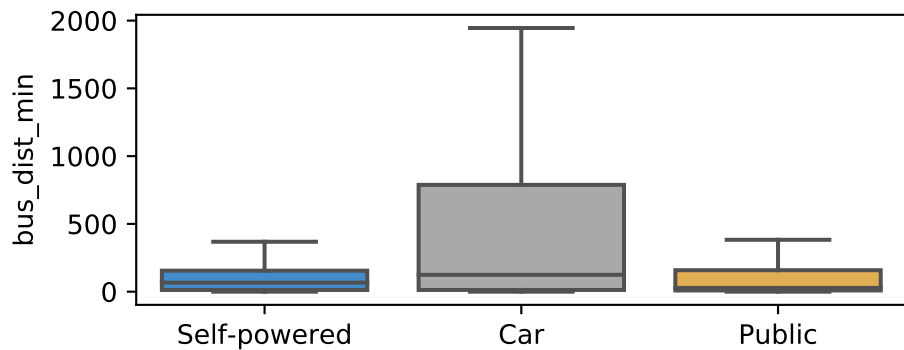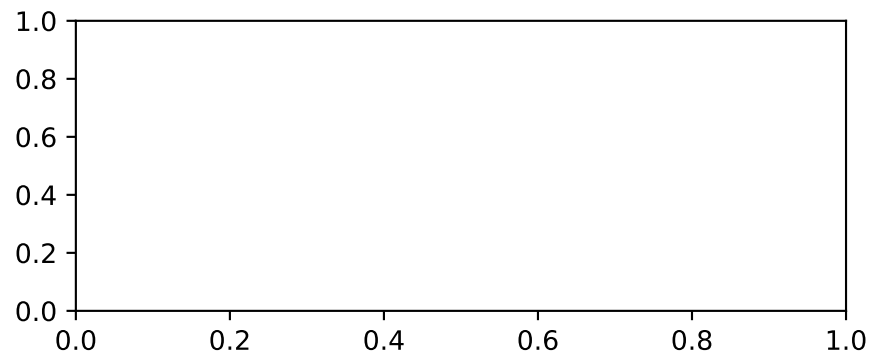

Supplement: S2 Fig — This figure presents box plots for the geolocation based features. The plot contains both direct features, i.e. velocity and acceleration, and features based on distance to various geopgrahic information. (PDF) [file pone.0234003.s004.pdf]

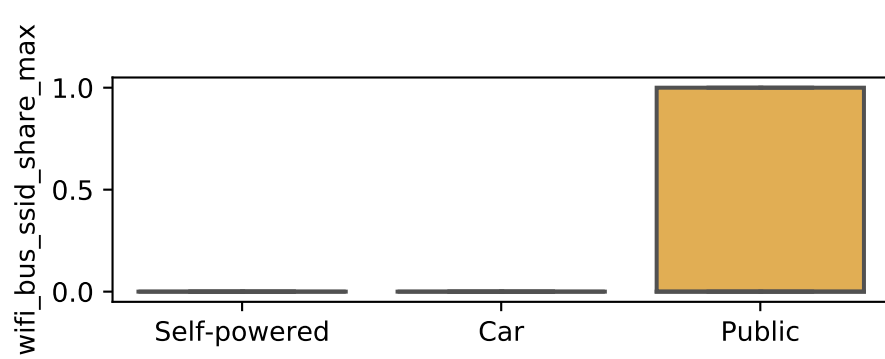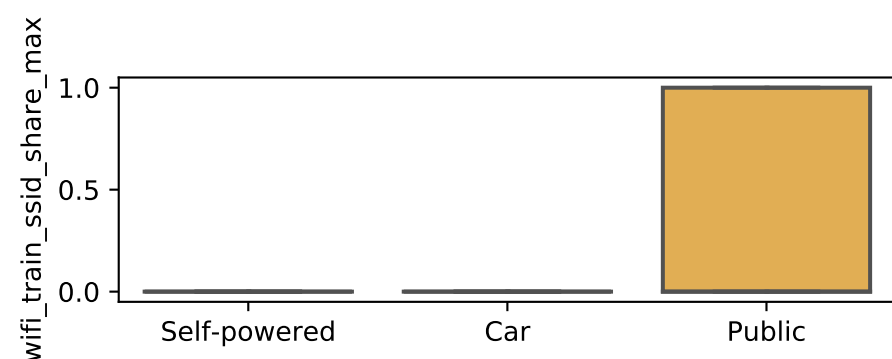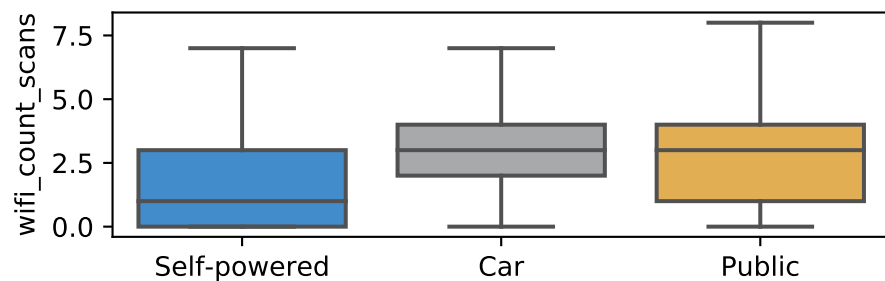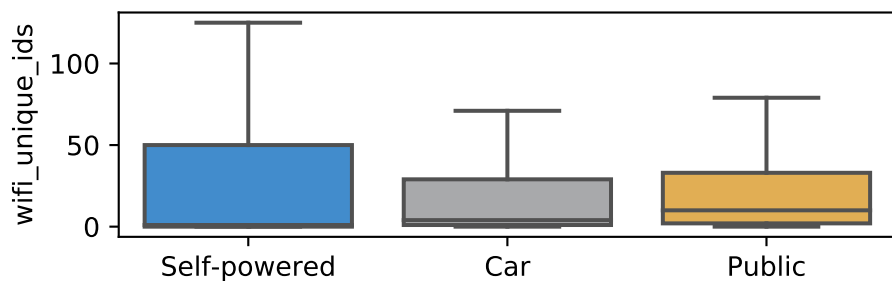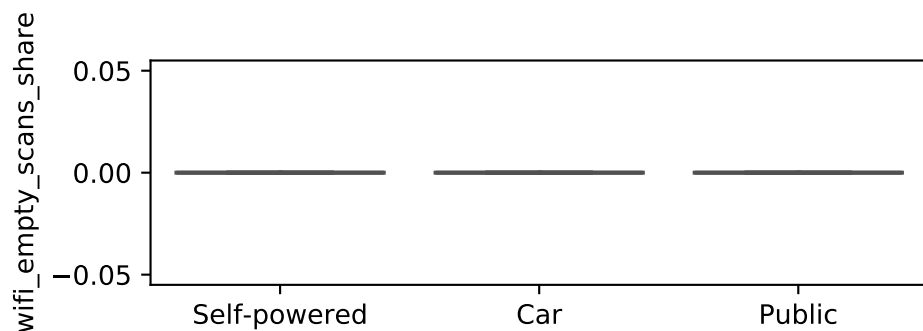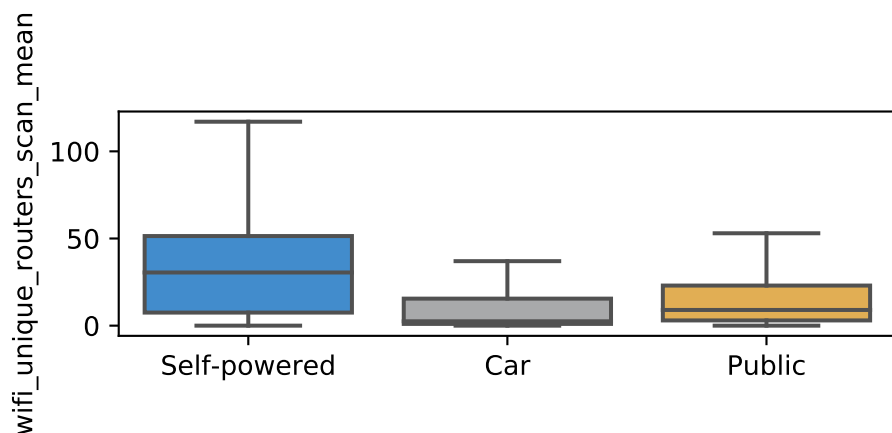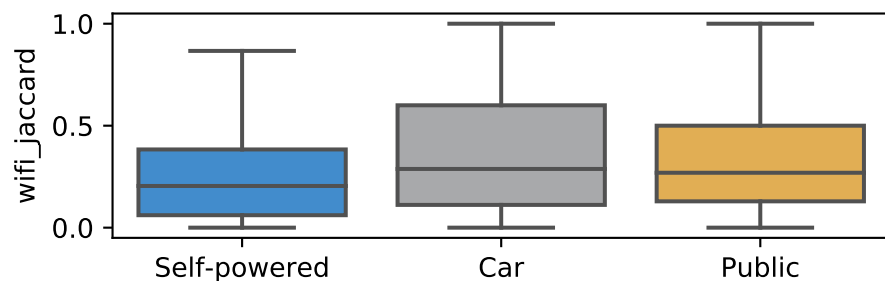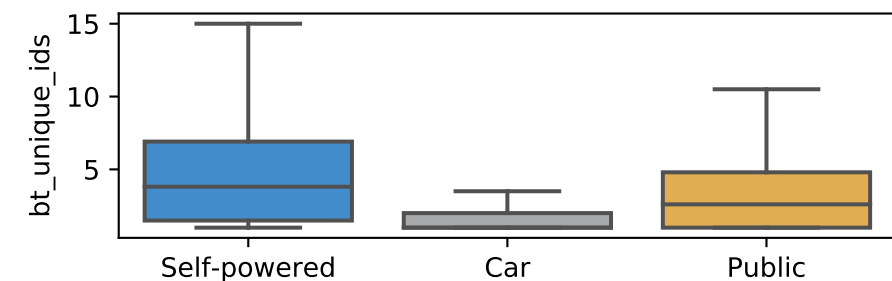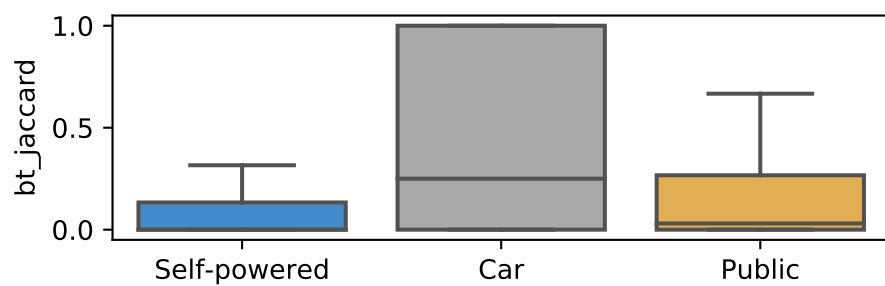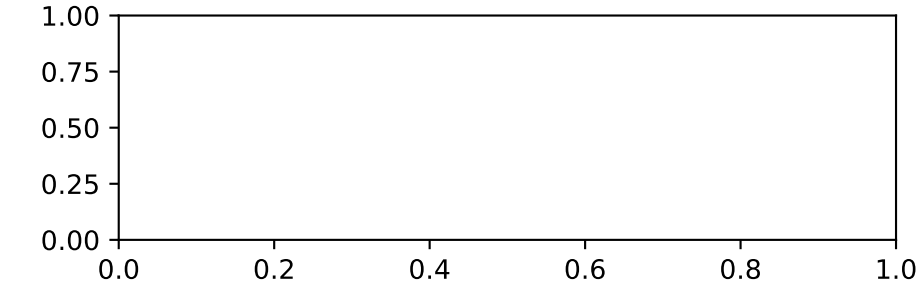

Supplement: S3 Fig — This figure presents box plots for the feature measured by Wi-Fi and Bluetooth features. The plot contains both features computed by investigating SSID for bus and train context as well as feature measuring the presence and change in Wi-Fi APs and Bluetooth devices. (PDF) [file pone.0234003.s005.pdf]
