# Supplementary material for: Inferring transportation mode from smartphone sensors: Evaluating the potential of Wi-Fi and Bluetooth
Source: PLoS One. 2020 Jul 2;15(7):e0234003. doi: 10.1371/journal.pone.0234003 (PMC7332005; doi:10.1371/journal.pone.0234003)
Supplement: S1 Table — This table contains all the mean performance metrics across all model specifications and feature sets investigates. The mean is computed using the resampled data 1,000 times into training data for model building and test data for evaluating the model. (PDF) [file pone.0234003.s006.pdf]

				F_1 score		Accuracy		Precision		Recall	
Model	Target	GIS context	Wi-Fi and Bluetooth	Segment	Minute	Segment	Minute	Segment	Minute	Segment	Minute
LR	3 modes	Included	Included all	0.699	0.716	0.825	0.770	0.801	0.757	0.658	0.700
RF	3 modes	Excluded	Excluded SSID	0.685	0.688	0.825	0.755	0.751	0.711	0.655	0.678
			Exluded all	0.565	0.599	0.763	0.694	0.641	0.627	0.543	0.591
			Included all	0.788	0.762	0.868	0.804	0.828	0.775	0.765	0.755
		Included	Excluded SSID	0.768	0.752	0.860	0.798	0.814	0.771	0.739	0.744
			Exluded all	0.749	0.718	0.851	0.765	0.814	0.732	0.713	0.711
			Included all	0.828	0.787	0.895	0.821	0.865	0.804	0.805	0.779
	5 modes	Excluded	Excluded SSID	0.590	0.558	0.807	0.721	0.715	0.594	0.549	0.547
			Exluded all	0.353	0.368	0.741	0.623	0.417	0.386	0.350	0.369
			Included all	0.733	0.705	0.860	0.789	0.823	0.733	0.694	0.694
		Included	Excluded SSID	0.780	0.741	0.868	0.793	0.835	0.755	0.757	0.741
			Exluded all	0.763	0.704	0.851	0.760	0.836	0.715	0.732	0.710
			Included all	0.823	0.785	0.895	0.819	0.871	0.799	0.799	0.786
SVM	3 modes	Included	Included all	0.697	0.706	0.825	0.760	0.784	0.739	0.678	0.697
