# Supplementary material for: Inferring transportation mode from smartphone sensors: Evaluating the potential of Wi-Fi and Bluetooth
Source: PLoS One. 2020 Jul 2;15(7):e0234003. doi: 10.1371/journal.pone.0234003 (PMC7332005; doi:10.1371/journal.pone.0234003)
Supplement: S2 Table — This table contains all the gain in model performance broken down by mode when excluding either Wi-Fi context feature or all feature based on Wi-Fi or Bluetooth. Associated with the gains we have computed the p-value. The F1 is a mean computed using the resampled data 1,000 times into training data for model building and test data for evaluating the model. (PDF) [file pone.0234003.s007.pdf]

		Wi-Fi context features		All features	
		Gain in F_1	p-value	Gain in F_1	p-value
Mode	GIS context feat.				
Car	Excluded	0.120	0.022	0.276	0.000
	Included	0.057	0.101	0.086	0.052
Public	Excluded	0.170	0.001	0.349	0.000
	Included	0.112	0.004	0.128	0.011
Self-powered	Excluded	0.015	0.032	0.044	0.000
	Included	0.011	0.077	0.022	0.012
