# Supplementary material for: Inferring transportation mode from smartphone sensors: Evaluating the potential of Wi-Fi and Bluetooth
Source: PLoS One. 2020 Jul 2;15(7):e0234003. doi: 10.1371/journal.pone.0234003 (PMC7332005; doi:10.1371/journal.pone.0234003)
Supplement: Attachment — Submitted filename: response to reviewers.pdf [file pone.0234003.s009.pdf]

Response to reviewers

Reviewer #1:

Recommendation:

Comments:

This paper proposes a classification system that uses Wi-Fi and Bluetooth data to infer the transportation behavior of mobile phone users. The usage of scanned Wi-Fi data is an interesting idea. However, there are some major issues should be addressed.

1. In this study, the purpose of transportation mode detection is not clear. In many recent studies, the aim of detection the transportation behavior or OD information of the public is to analyze the transportation patterns, problems or development trend of a city. Therefore, a large amount of user data is employed in these studies. Differently, in this study, the proposed method is mainly used to detect and classify the transportation behavior of personal users. The motivation of this study is not clear. We agree that the sample size will grow bigger if more users can install the app in their mobile phone. However, in practice, it is very difficult to collect enough data for urban problem analysis by using apps with few users. Compared to other urban transportation data, e.g. taxi trajectories data or public smart card data, the collection of user Wi-Fi or Bluetooth data is difficult because it require the public to install particular apps on their phones. The practicability of this study should also be considered.

> This is a useful comment which we have used to revise our paper (see also references below). We have added a discussion about the practicability of the study. In particular, we now discuss pre-labelled data from, e.g. from taxi-trajectories, rented bikes and smart cards for public transport.

2. Similarly, the title of this study is not specific or detailed. “Urban transportation sensing” is a very big issue. The scope of this study should be narrowed to make the study purpose more clear.

> We thank the reviewer for pointing this out. To address this we have changed the title of the paper to “*Inferring transportation mode from smartphone sensors: Evaluating the potential of Wi-Fi and Bluetooth*”, which directly addresses the purpose of the paper.

3. Another major problem is that other urban transportation sensing methods have not been considered in this study. Recently, there are many studies that use urban big data, e.g., taxi trajectories data, public smart card data or mobile phone data, to analyze urban transportation

patterns. These studies have not been considered and included in the literature review in this study. For example:

Wei Tu, Rui Cao, Yang Yue, Baoding Zhou, Qiuping Li, Qingquan Li. Spatial variations in urban public ridership derived from GPS trajectories and smart card data. Journal of Transport Geography. 2018, 69, 45-57

Although our paper focuses on measuring individual-level transportation behaviors, we have added three references that highlight the complementary macro-level perspectives provided by smart cards.

- Gan Z, Yang M, Feng T, Timmermans H. Understanding urban mobility patterns from a spatiotemporal perspective: daily ridership profiles of metro stations. Transportation. 2018 May 1:1-22.
- Wei Tu, Rui Cao, Yang Yue, Baoding Zhou, Qiuping Li, Qingquan Li. Spatial variations in urban public ridership derived from GPS trajectories and smart card data. Journal of Transport Geography. 2018, 69, 45-57
- Weng J, Tu Q, Yuan R, Lin P, Chen Z. Modeling mode choice behaviors for public transport commuters in Beijing. Journal of Urban Planning and Development. 2018 May 25;144(3):05018013.

4. Most of the papers have been cited for literature are before 2016. It would be better to add some more papers of 2016-2019, but don't increase the volume of current literature.

Good point, we have updated our literature review and discussion pertinent recent papers to replace older references.

- Gadziński J. Perspectives of the use of smartphones in travel behaviour studies: Findings from a literature review and a pilot study. Transportation Research Part C: Emerging Technologies. 2018 Mar 1;88:74-86.
- Wang Z, He SY, Leung Y. Applying mobile phone data to travel behaviour research: A literature review. Travel Behaviour and Society. 2018 Apr 1;11:141-55.
- Welch TF, Widita A. Big data in public transportation: a review of sources and methods. Transport Reviews. 2019 May 11:1-24.
- Carpineti C, Lomonaco V, Bedogni L, Di Felice M, Bononi L. Custom Dual Transportation Mode Detection by Smartphone Devices Exploiting Sensor Diversity. In2018 IEEE International Conference on Pervasive Computing and Communications Workshops (PerCom Workshops) 2018 Mar 19 (pp. 367-372). IEEE.

5. The method section (section 3 and 4) is too descriptive.

We have now added a more critical analysis of the methods, e.g. pointing to other powerful supervised learning machine learning algorithms, discuss pre-processing steps.

6. In section 3.2, the estimation and collection of the location of Wi-Fi routers in a city is very important for the proposed method and should be detailed described in the main text.

This text has been moved from the appendix to the main text as part of data description.

7. In section 5, it lacks a comparison between the proposed method and other methods (e.g., using smart card). The discussion about different transportation sensing methods is also necessary.

Good point. We now include a concise comparison of our individual-level Wi-Fi transportation mode detection method to macro-level transportation sensing methods (smart card). The gist of the comparison is the following: our Wi-Fi method requires users to make use of an app (which collect raw data at certain frequencies) whereas smart card systems require users and transportation modes to adopt the smart card system. Smart cards can only track individual behavior across certain included modes but not others (biking and self-powered transportation modes frequently do not participate in smart card systems).

8. In fig 2 and fig 8, the scale of the map as well as the name of the regions is missing.

We have added scales and labels for the Copenhagen and DTU area to Figures 2, 8.

9. The transportation mode classification approach is running on a PC or a smartphone? More information should be provided?

It is running on a PC, this has been changed in the revised methods section.

Reviewer #2:

This study is an extension of a previous work published in a conference proceeding. The extension is stated be to infer location, identify some transportation modes, and derive transportation related features. This is interesting and meaningful.

We thank the reviewer for this comment. We want to clarify that the content of this article is novel and has not been published in a conference proceeding earlier. It is correct that we use a procedure developed in an earlier conference proceeding for mapping Wi-Fi routers to location (which is also outlined in the data description), however, the research question is novel. We have made this point more clear in the main text.

However, the writing of the paper is a big problem. I am even not sure what is the contribution of the paper because of the poor writing. Specifically, the title is “Wi-Finding: Urban transportation

sensing with smartphone Wi-Fi sensors". It seems from the title that a method or a device called Wi-Finding was developed. However, from the introduction, readers know an approach has been developed to infer location, identify some transportation modes, and derive transportation related features.

Thank you for pointing this out. To address this we have changed the title to "*Inferring transportation mode from smartphone sensors: Evaluating the potential of Wi-Fi and Bluetooth*". The new title is consistent with the introduction.

It is noted in Lines 53 – 54 that Section 4 is an overview of the proposed method. However, the heading of Section 4 is "classification approach". Therefore, it is not clear where the extension was explained. Is the "classification approach" is the core of this paper?

Yes, the classification approach is the extension. We have clarified this in the paper outline where the "classification approach" is called "method" (lines 53-54).

The 4th sentence in the abstract reads, "Our approach yields an overall accuracy ...". What is the "approach"? This "approach" should be more clearly explained in the first 3 sentences.

Thank you for this comment, we have added two sentences to the abstract to explain this more carefully.

There is a gap between the 2nd and the 3rd paragraphs of the introduction. In the 2nd paragraph, it is noted that Wi-Fi and Bluetooth traces are not widely used. Therefore, in the 3rd paragraph, this study will use Wi-Fi and Bluetooth traces. However, there are a lot of devices that are not widely used.

Good point by the reviewer. Our argument is the following: WiFi and Bluetooth are not widely used for mode of transportation detection. We show that they are highly useful for this task. These signals are also widely available to modern smartphones. Therefore our research shows a way to improve upon state-of-the-art. We have updated the main text to make this point more clear.

Line 36. What are the "some of the transportation modes" refer to? Line 39. What are the "transportation related features" refer to?

We have clarified these points in the introduction of the manuscript.

The second section presents a literature review of related works. What are the problems of these works? This significance of this current work would be limited if the related words are good enough.

We are not sure we completely understand this particular point. We identify several areas where existing transportation mode detection approaches might be improved and

highlight the limitations of related work using Wi-Fi traces. We also mention that the approach we use in this paper is tailored towards transportation science applications where either further discrimination between public and other modes is desired or accelerometer data is unavailable.

The third section is about data collection. This section should come later than the method section, if the method is independent of data collected.

We have changed the order of the two sections..

Reviewer #3:

This paper proposes a mobile phone-based transportation model detection method. It's very interesting. I have some questions about feature extraction. The authors wrote "Step 2 segments trips using long and short stops". How to determine the long and short stops?

Good point. The process of computing stops is as follows. First, long stops are computed using low frequent location provided through Android at the five minute level. The raw location is likely to be more robust so we wanted to compute those first. Second, we compute short stops in the gap between long stops using the the location data at the minute level which consists of the combined raw data and Wifi-inferred location data.

We have clarified the process for and motivation of computing long and short stops in the manuscript.

After we get the trip segment, how to extract the features?

We thank the reviewer for pointing out the sparsity of details. The features were extracted at the minute level. Before that low frequent data (5m) was then converted to higher frequency by interpolating the missing observations while the high frequent Wi-Fi measure (15 sec) was converted to lower frequency by aggregation.

This point has been made clear in the revised manuscript.

I think the length of the trip segments are different?

Yes, trip lengths can be different. The model predictions are, however, made at the minute level and then aggregated. We have clarified that in the manuscript.

“To calculate the features, we must use a sliding window to process the trip segments.” How to determine the length of the sliding window?

The sliding window is depends on whether it is short or long stops. The algorithm we used detects whenever a person whenever a person remains within a certain radius over

a given period of time (5 min for short stops, 15 min for long stops). Once it has processed some data it continues to the next available data. We have made this point more clear in the text.
