# Supplementary material for: Inferring transportation mode from smartphone sensors: Evaluating the potential of Wi-Fi and Bluetooth
Source: PLoS One. 2020 Jul 2;15(7):e0234003. doi: 10.1371/journal.pone.0234003 (PMC7332005; doi:10.1371/journal.pone.0234003)
Supplement: Attachment — Submitted filename: response to reviewers.pdf [file pone.0234003.s010.pdf]

Response to reviewer comments

Reviewer #1

This paper uses information from Wi-Fi access points and Bluetooth devices, enhancing GPS and geographic data to improve transportation detection on smartphones through machine learning approaches. It is a timely study with promising future applications due to the prevalence of wi-fi, Bluetooth, and smartphones. The results are well explained. However, the methodology part is confused. There are some comments for authors to improve the paper.

> We appreciate the reviewer's summary, appraisal of our paper's relevance and suggestions for improvement.

1. In 2 Related Work section, for travel mode inferring, another type of research encompassing GIS application is using map service API, such as Google Route API, to provide API travel path/route for mode detection. As you mentioned Google Location API, more about those should be explored. You may refer to those research articles, and one example likes this:

a. Zhu, Lei, and Jeffrey D. Gonder. "A driving cycle detection approach using map service API." Transportation Research Part C: Emerging Technologies 92 (2018): 349-363.

> Thank you for the suggestion! We have added a number of new references to the paper, including the one listed above.

2. In 3 Methods section, "We limited the number of classification models to three:" Why you choose those three methods? A little explanation may need here. Also, later, the random forest (RF) has been selected for analysis. A more detailed introduction of RF and its basis are necessary. I suggest adding some content about the RF basis.

> We have clarified that our choice of models was based on existing research which we now cite in the main text.
>
> We have also added more details on the estimation of decision trees in the main text, which already contained a description of RF in the original submission.

3. In 3 Methods section, what are segment level and minute level? Please define them in the context. Also, why the segment level is selected?

> We have added a detailed explanation of which parts are segment level and which parts are minute level.

We chose the segment level because this was our unit of analysis and is the more prevalent in the literature. Further, understanding the transportation behavior at the segment-level also has practical utility. We have updated the text to reflect this more clearly.

4. In Figure 2, it is hard to read that DTU is an orange dot, not a blue dot. Also, for Copenhagen, it is in gray or orange?

Well spotted, thank you. The mismatch between the text and figure has been fixed.

5. On page 8, “The app collected location, Bluetooth, and Wi-Fi as detailed in [68]” Please explicitly explain the data collected. How does it look like roughly?

A brief description of each data type has been added.

6. Page 9, “Subsequently, we identify router sightings, associating Wi-Fi routers to locations where they were scanned” What are router sightings? What is a Wi-Fi router? A picture or diagram of those or of the whole procedure of estimate the location routers may be helpful.

The text has been edited and now uses the terminology of scanned routers (which was also expanded in response to comment 5 above)

7. For segmentation on page 10, other literature on determining stationary time interval may need.

Our choice of minimum threshold for stationary time has now been described in greater detail with references to the literature on stop locations.

Reviewer #2:

This paper studies an interesting problem of identifying the travel mode by using Wifi and bluetooth data. Comprehensive experiments have been conducted to show the effectiveness of using Wifi and bluetooth data for travel mode identification. However the motivations of choosing LR, SVM and RF are not clear. Why do you limit the classifiers to those three?

We have clarified that our choice of models was based on existing research which we now cite in the main text. We limited the number of models as these three represent different kind of models (respectively linear, kernel and tree based). We omitted neural networks as they require a lot of data and customization in order to work.

The other concern is if the temporal resolution of the signal influence the performance. Some experiments should be performed to show the influence of the temporal resolution of the signal.

This is a good point. We have performed an experiment to assess the influence of the temporal resolution. The results from the experiment have been inserted into Appendix 2 and are now referenced in the main text. I aggregated the temporal to the five minute level (from one minute) and provided similar results to the main analysis. The experiment provided more or less comparable performance - compared with the main specification the 5-minute model yielded a slightly higher F1 score (0.85 up from 0.83) but slightly lower accuracy (0.88 down from 0.90). Moreover, they yielded the same conclusions in terms of model performance, although with slightly lower statistical significance.
